# Long-Term Evaluation of Tooth Transplantation: An Umbrella Review

**DOI:** 10.3390/jcm13113341

**Published:** 2024-06-05

**Authors:** Mohamed Jaber, Prathibha Prasad, Mohammad Akeil, Abdulrahman Moufti, Almustafa Al-Sammarraie, Chuaeib Charaf Eddin

**Affiliations:** 1 College of Dentistry, Center of Medical and Bio-Allied Health Sciences Research, Ajman University, Ajman P.O. Box 346, United Arab Emirates; 2 Department of Oral Pathology, Saveetha Dental College and Hospitals, Saveetha Institute of Medical and Technical Sciences, Saveetha University, Chennai 600077, India

**Keywords:** autotransplantation, teeth transplantation, primary teeth, permanent teeth, umbrella review

## Abstract

**Aim/Objective**: This umbrella review of systematic reviews aims to summarize the available data regarding both success and survival rates of tooth autotransplantation, in addition to analyzing the risk factors that are connected to those rates. **Methods:** This umbrella review was performed according to the evaluation of various meta-analyses and systematic reviews following AMSTAR2 guidelines. A systematic search of literature on PubMed, Scopus, MEDLINE, EMBASE, and the Cochrane Database. Six systematic reviews were included. Explicit inclusion and exclusion criteria were applied. It is registered in PROSPERO under the registration number (CRD-42023415623). **Results:** The studies reviewed were written from 2014 to 2018, which extracted the information from various studies spanning from 1968 to 2014. According to the selected studies regarding autotransplanted teeth in humans, they showed the following: A survival rate overall of 87.39% and a success rate overall of 90.29%. These factors were the most common in relation to the success of the autotransplanted teeth: age, gender, and stage of root development. On the other hand, common unfavorable results linked to the transplanted teeth in these studies were failure, ankylosis, and internal root resorption, followed by extraction and hypermobility. **Conclusions:** The wide body of evidence gathered illustrates that autotransplantation is an operation that dispenses high rates of survival and success. Furthermore, risk factors like root development stage, recipient site, and donor tooth type established a remarkable association with the negative outcomes of the procedure. For successful tooth autotransplantation, patient selection is crucial. Younger patients and those with donor teeth at an optimal stage of root development typically experience better outcomes. Preoperative planning should include comprehensive evaluations and advanced imaging techniques to accurately assess both the donor tooth and the recipient site. Nonetheless, on account of heterogeneity and the quality of the studies included in this investigation, caution should be taken when interpreting the mentioned results.

## 1. Introduction

A tooth is transplanted from one place in the oral cavity to another in the same mouth during a dental treatment, which is referred to as tooth transplantation [1]. Restoring the teeth’s function and esthetics is the primary objective of tooth transplantation [2]. Increasing awareness of the advantages and developments in dental technology has resulted in an increase in the frequency of tooth transplants in recent years. For people who are missing teeth as a result of trauma, pathologies, or other reasons, the process may be a safe and efficient alternative [3]. 

Tooth transplantation is typically performed in cases where a patient has a missing or damaged tooth that cannot be restored using other dental procedures, such as dental implants or dentures. The procedure is often performed on young patients who have lost a tooth due to trauma or developmental defects. Teeth transplantation is also sometimes used in cases where a patient has lost multiple teeth and is not a suitable candidate for dental implants or dentures [4].

The age and general health of the patient, as well as the location of the implanted tooth, all affect the success of transplantation treatments. The range of success rates for tooth transplants is 50–90%. Tooth transplants from living donors have been found to have a higher rate of success than those from cadavers [5]. Positive results for tooth transplantation procedures have been documented in various studies. According to a 2009 study by Kirsch et al., 90% of children and adolescents who underwent tooth transplants achieved successful results [6]. 

Although long-term success is achievable, problems can arise. Kirsch et al. found that infection accounted for 9% of tooth transplantation-related issues [6]. In addition, 2% of cases suffered rejection. Yanik et al. found in another study that 4% of cases showed root resorption [5]. When the recipient’s immune system targets the implanted tooth’s root, root resorption takes place, which results in tooth loss [7]. The causes of failure of the autotransplant is chronic root resorption, inflammatory resorption, replacement resorption i.e., ankylosis, marginal periodontitis, apical periodontitis, caries, and trauma [8]. Though there are times when problems from tooth transplantation are unavoidable, there are precautions that can be taken to lessen the possibility of their occurrence. Careful monitoring and proper patient selection are some of the most important actions of prevention [9,10,11]. 

The purpose of the current review is to gather the information that is currently available concerning the success and survival rates of tooth autotransplantation and explore the risk factors associated with them.

## 2. Materials and Methods

The current review was made on the basis of a report assessing many systematic reviews and meta-analyses following the AMSTAR guidelines and was given the registration number CRD-42023415623 on the International Prospective Register of Systematic Reviews (PROSPERO). Furthermore, AMSTARII was used to assess the systematic reviews’ methodological quality; it is a reliable, valid, and efficient measurement instrument for evaluating numerous systematic reviews [12].

Aim: This umbrella review of systematic reviews aims to summarize the available data regarding both success and survival rates of tooth autotransplantation, in addition to analyzing the risk factors that are connected to those rates.

**Hypothesis 1**:  *Younger patients (under 18 years old) exhibit higher success and survival rates in tooth autotransplantation compared to older patients.*

**Hypothesis 2:**  *Donor teeth with incomplete root formation demonstrate significantly better outcomes in autotransplantation than teeth with fully formed roots.*

### 2.1. Search Strategy 

The systematic review was defined in line with the Cochrane Collaboration and the Preferred Reporting Items for Systematic Reviews and Meta-Analyses (PRISMA) Statement [13]. High-quality systematic reviews were included that stated the long-term outcomes of tooth autotransplantation in humans.

To recognize reviews that comply with the inclusion criteria, the databases that have been searched are as follows: PubMed/MEDLINE, Scopus, Google Scholar, and Springer. A manual search was also conducted on journals included in the acquired research studies’ respective reference lists. For the purpose of this search, the terms of the Medical Subject Headings (MeSH) chosen included (autotransplantation OR (autologous AND transplantation)) AND (tooth OR teeth OR incisor OR canine OR cuspid OR bicuspid OR premolar OR molar OR dent OR wisdom teeth OR wisdom tooth), PICO, which stands for P (Patient Population), I (Intervention or Exposure—in the case of observational research), C (Comparison), and O (Outcomes), was utilized. For this umbrella, the PICO approach included Population (Patients undergone autotransplantation), Exposure (Teeth extraction), Comparison (Different types of teeth transplantation) and Outcome (outcomes of outtransplantation). 

### 2.2. Eligibility Criteria

For acquiring research studies that directly connect to the focus of this study, the following eligibility criteria were put in place: studies of full-text articles needed to be available, not just in an abstract, brief form. Moreover, they needed to utilize a systematic review or systematic review and meta-analysis design, which aims at all age groups (both children and adults). A data collection form was used to gather data for each of the included studies, which comprised surgical procedure, study interval, number of patients, the study’s aim, quality assessment, and the conclusion of the studies. Studies with the following characteristics were excluded: studies without follow-up, non-English language studies, any non-systematic review or meta-analysis studies, studies reporting teeth transplantation in animals, and other reasons, such as case studies and insufficient data.

Two authors (M.A. and A.A.) independently evaluated the quality of the studies included. Two authors (A.M. and C.T.) separately did the data extraction from the prespecified data extraction sheet in Microsoft Excel after ensuring the quality of each study. The fifth author (P.P.) was responsible for the final decision on the inclusion or exclusion of the article. The variables extracted from each qualifying study were: first author’s name, publication year, study length, location of study, design of this study, median follow-up time, source of data, and size of sample. The AMSTAR 2 Quality Assessment of Systematic Reviews was used to assess the risk of bias in all the articles included in this study, as follows: (a) PICO Question, (b) aim of this study, (c) study design selection explained, (d) comprehensive literature search strategy, (e) were the included studies described in adequate detail, (h) was there a satisfactory technique used to assess risk of bias, and (f) was the source of funding for the studies reported and appropriate statistical methods performed in case of meta-analysis 

According to the risk of bias analysis assessment that was conducted, the included studies that were chosen for further analysis had an overall quality that was estimated to be low (i.e., AMSTAR 2, 4 studies scored low (between 0 and 4), 2 were of moderate quality (5–8), and 0 were of high quality (9–11).

The critical evaluation of the accuracy of systematic review findings was performed using the Risk of Bias in Systematic Reviews (ROBIS) tool, which assessed the quality and potential for bias (Table 1). Two independent investigators (A.M. and C.T.) evaluated each systematic review by using the ROBIS tool, which consists of three phases, which consisted of determining relevance, identifying issues with the review procedure, and assessing the risk of bias in the review. The second phase of the ROBIS tool involved four domains: study eligibility assessment (Domain 1), identifying and selecting studies (Domain 2), evaluating selected data and study quality (Domain 3—risk of bias), and synthesizing and analyzing findings (Domain 4), all of which were crucial to minimizing any potential bias. Using the four domains’ interpretation, it was decided whether there was a low, high, or unclear risk of bias.

Quality assessment and assessment of risk of bias: an assessment of the validity of the results presented in systematic reviews was critical for the recommendations and was performed using the Risk Of Bias In Systematic Reviews (ROBIS) tool. Each systematic review was assessed independently by two investigators (A.M. and C.T.) using the three phases of the ROBIS tool: (A) assessment of relevance, (B) identification of concerns with the review process, and (C) judgment of the risk of bias in the review. The second phase involves four domains: assessment of study eligibility (Domain 1), identification and selection of studies (Domain 2), data selection and study appraisal (Domain 3—risk of bias), and synthesis and findings (Domain 4) critical to the minimization of bias. The risk of bias was determined as low/high/unclear based on the interpretation of the four domains.

### 2.3. Inclusion Criteria

The searches yielded 6 studies. These reviews’ titles and abstracts were checked. The six reviews that were potentially appropriate were used to obtain and score the full-text English articles. If the following selection criteria were satisfied, systematic reviews were included:

The reviewers used certain criteria to determine which primary studies should be included and excluded. 

The review results, success rate, and prognosis of the transplanted teeth were reported with odds ratios and effect sizes.

A.M. and C.T. screened titles and abstracts, and any disagreement was resolved through further discussion. For further assessment, full-text articles were retrieved.

One reviewer (M.J.) gave each of the six reviews a score, and one of two other reviewers (M.A. and A.A.) gave an independent score using the data extraction form. There was a 94% inter-rater agreement. Discussion was used to settle 6% of cases where there were disputes. Six systematic reviews in all were included in this study after meeting all inclusion requirements.

### 2.4. Exclusion Criteria

This study did not contain descriptive reviews. 

Reviews related to tooth transplantation on animals were excluded.

### 2.5. Quality Assessment

The reviews that meet the inclusion criteria for quality assessment were undertaken by A.M. and C.T. if at least five of the criteria required by AMSTAR 2 were satisfied [12]. A verified measurement tool for evaluating the quality of systematic reviews was included. For example, some of the AMSTAR-2 quality criteria evaluate whether “a priori” design was established, duplicate study selection and data extraction were included, the literature search was comprehensive, whether the quality of primary studies was examined, etc.

### 2.6. Data Extraction and Analysis

#### Data Extraction

Data extraction was accomplished by M.A. and A.A. by means of the form of standardized data extraction. The following features of the reviews were evaluated using the form: objectives, reviewed studies number, the primary studies’ number, type of teeth transplanted, range of primary studies follow-up period, the literature searches period, sample characteristics, sample size, recommendations for future research, and limitations of primary studies and review. 

The full texts of the chosen articles were evaluated. Using the Cohen ĸ coefficient, inter-reviewer agreement was measured to determine study selection. The Landis and Koch scale was used to determine the degree of agreement, where kappa values <0 meant there was no agreement; agreement ranges from 0 to 0.20 being minimal, 0.21 to 0.40 being reasonable or fair, 0.41 to 0.60 being moderate, 0.61 to 0.80 being substantial, and 0.81 to 1 being perfect. Disagreements regarding discrepancies were settled by conversation with a third reviewer. Disagreements were resolved through discussion.

The electronic search was performed using the keywords autotransplantation, dental, tooth, teeth, incisor, canine, cuspid, bicuspid, premolar, molar, and wisdom tooth, and modifications were followed for each unique database. Regarding the studies’ status and year of publication, there were no restrictions. Lastly, manual searches were conducted on the reference lists of the articles deemed suitable for inclusion in the meta-analysis.

## 3. Results

**Figure 1:**
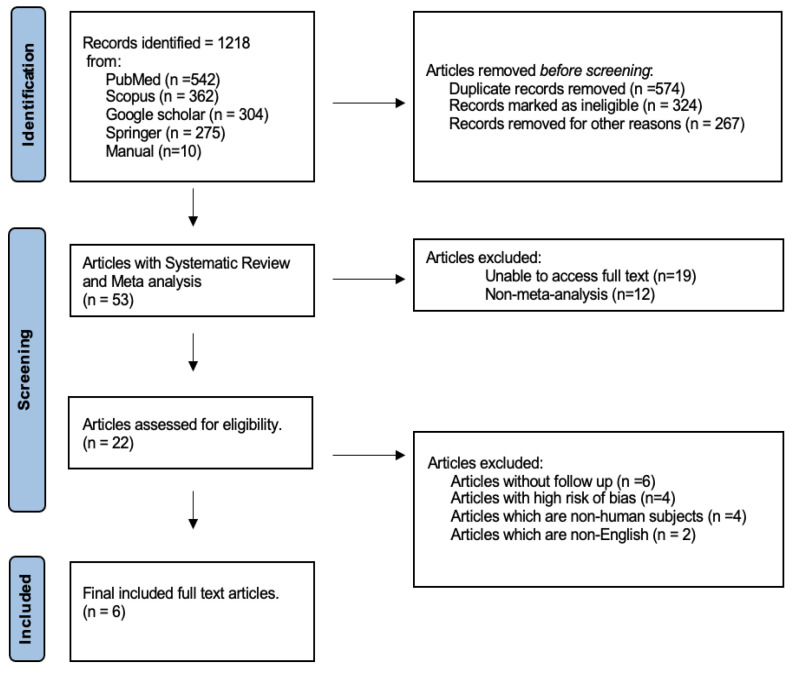
PRISMA CHART.

Through digital search, at first, 1218 records were retrieved. Then, by manually examining the full text articles’ reference lists, 10 more studies were added. For inclusion, a selection of six studies was made to evaluate the success and/or survival rate of tooth autotransplantation in human subjects (Figure 1). According to the general caliber of the included studies, the risk of bias analysis was overall low (Table 2) [13].

### 3.1. Characteristics of the Included Systematic Reviews 

The systematic reviews’ studies’ time periods varied from 2000 to December 2022. Cohort, case-control, or cross-sectional studies made up most of the primary studies, which were all observational studies (Table 3). Only English-language studies were included, and PubMed was the most frequently searched database.

### 3.2. Synthesis of Results

Out of the six systematic reviews found, four offered a quantitative synthesis of the data, whereas the other two did not. The following factors served as the basis for the qualitative analysis overall: Table 4.

Success and Survival rates.

Patient’s age and gender at the operation’s time. 

Stage of root development.

Autotransplanted tooth donor’s site. 

The recipient site for the autotransplanted tooth. 

Type of donor tooth—canine or premolar.

Surgical technique.

The healing period fixation splint type used. 

Orthodontic forces applied to the autotransplanted teeth. 

### 3.3. Success and Survival Rates

In the autotransplanted teeth of human subjects, an overall success rate of 90.29% and an overall survival rate of 87.39% were achieved, according to the selected studies. 

Autotransplantation survival and success rates can be affected by many factors, such as the patient’s age and gender, root development stage, recipient and donor sites, type of donor tooth, surgical procedure, type of fixation splint, and orthodontic force implementation, which were investigated in only 6 studies. It was found that there were significant associations between (a) the autotransplanted teeth’s stage of root development at operation time, root resorption, and the appearance of an open apex and ankylosis, pulp necrosis, necessity for extraction, and the outcome with the greatest failure in each situation; (b) the transplanted teeth’s recipient site, more precisely when the same site is used, and pulp necrosis; and (c) in each study, the type of donor tooth with an outcome that presented the worst failure, when molars rather than canines were used as transplants, and the occurrence of pulp necrosis when second premolars were used as a replacement for first premolars.

Based on the six included studies, for autotransplanted teeth, the following factors were most frequently associated with success and failure: 

Patient’s age on procedure day—more/less than 20 years of age.

Patient’s gender. 

Donor tooth root development stage—(open/closed) apex. 

Donor site of transplant—maxilla or mandible. 

Recipient site of transplant—(same as/different from) donor site.

Type of donor tooth—canines vs molars and 1st vs 2nd premolars 

Surgical method used—osseous graft used or not.

Type of splint used for transplant fixation— (rigid/suture) splint type. 

Orthodontic force implementation on the transplanted teeth.

Moreover, according to these studies, for the transplanted teeth, the most typical unfavorable outcomes that might be linked with the mentioned risk factors were as follows:

Root resorption.

Ankylosis.

Extraction. 

Failure. 

Hypermobility. 

Pulp necrosis. 

Pulp obliteration. 

### 3.4. Description of Studies and Baseline Characteristics 

Among the studies, the number of patients used varied, with more than 3000 patients being the mean study group size of the studies included. The mean age of the patients was reported to be over 20 years. In addition to this, younger adults were used in several of the studies.

Molars were the most used teeth for autotransplantation as well as premolars [14,15,16,17,18]. Incisors appeared as transplants in four studies [14,16,17,18]. Most of the research mentioned used a consistent surgical protocol. The most frequent technique entailed contouring the recipient socket with a surgical bur when necessary to reduce bone and reshape the socket prior to transplantation. In other studies, split osteotomies and the use of bone transplants were also employed when the recipient region’s bone volume was insufficient. There have also been reports of other extra treatments to speed up the process, such as the creation of tooth replicas or the use of culture medium for transplants. The direct method was applied in most cases (Table 5). 

The use of various fixations, such as suture splints and/or flexible and rigid splints, was also noticed. The two forms of fixation were used equally across the studies, with suture splints used in more studies. Furthermore, the follow-up period of the studies ranged from 6 months to almost 26 years, indicating a large variation. 

Inflammatory and replacement root resorption, pulp necrosis, and decreased root formation were the most frequent complications connected to the loss of transplanted teeth and/or their failure. Nevertheless, the factors were not examined in the same way in all studies. Ankylosis was the most common complication noted in the studies that were considered (n = 5). Additionally, the same number of studies observed both root resorption and reduced or halted root development (n = 5). Another common observation was periodontal issues such as gingivitis, loss of attachment, and deeper periodontal pockets (n = 1). 

According to Chung, the survival rates ranged from 30.4% to 100% [14]. In contrast, success rates had a smaller range, from 57.5% to 96.5% in some cases [16,18]. The characteristics of the included studies used for analyzing the factors affecting the failure rates of autotransplantation are displayed in Table 6. In total, 4428 teeth—including 539 molars, 1787 premolars, 1229 canines, and 873 incisors—were autotransplanted in both jaws of the patients, with a mean age of 41.3 years (range: 6.6 to 76 years). Most teeth had an open apex. The follow-up period for all groups was anything between 6 months and 26 years. 

## 4. Discussion

In this study, the results showed a success rate ranging from 90.29% to 100%, which indicates that the survival of the transplanted teeth was affected by a range of factors. The survival rate was reported to be between 30.4% and 100%. The rate of internal/inflammatory resorption varied from 0 to 75.8%. The root development was reported to be the same as the donor tooth type, which was complete in all cases. That finding’s reliability would be compromised, though, as the six analyzed studies showed an increased degree of variability.

The success of the transplantation depends on various factors like donor tooth type, root formation, surgical protocol, splinting method, splinting material, and duration of the splint. Chung et al. conducted prospective and retrospective cohort studies and case series. The studies included 1261 patients, with no mention of the number of teeth, group of teeth, age range, and gender. Third molars were taken as donor teeth from the maxilla and mandible that had developed their roots completely and were at least five years old. The indications for transplantation were not mentioned. The follow-up period was not mentioned in all studies. The success rate ranged from 30.4% to 100%, and the survival rate ranged from 0.0% to 75.8%. The internal/inflammatory resorption rate ranged from 0% to 100% [14]. The root development was the same as formation, that is, complete. The surgical protocol was the same as that proposed by Nethander and Gault & Warocquier-Clerout [19,20]. The splinting method was suture splinting or wire splinting, and the splinting material was a thermoplastic retainer. The duration of the splint ranged from 0.5 to 61 weeks. The transplantation of maxillary central incisors had a follow-up of at least four years. The study’s orthodontic treatment was not mentioned. One study included all patients with advanced periodontitis.

Grisar et al. conducted prospective, retrospective cross-sectional studies and case series. The studies included 370 patients with 710 teeth, but not all studies included the number of patients. The group of teeth was not mentioned. The age range was 11 to 76, and both males and females were included. The donor teeth had complete/incomplete root formation and were two to five years old. The follow-up period was not mentioned. The success rate was not mentioned, but the survival rate ranged from 62% to 100%. The internal/inflammatory resorption rate was blank in some studies and ranged from 3.2% to 100%. The root development was the same as the formation. The surgical protocol was standardized, and the splinting methods were sutures, orthodontic wire, a plastic vacuform splint, and a metal cap splint. The studies did not mention orthodontic treatment, periodontal problems, or pulp necrosis [9].

Almpani, Papageorgiou, & Papadopoulo conducted a pragmatic clinical trial that included 386 patients with 413 teeth. The group of teeth was 345, and the age range was less than 20. Both males and females were included, and the donor teeth were permanent with complete root formation. The follow-up period was six months to one year. The success rate was 85.6%, and the survival rate was 93.9%. The internal/inflammatory resorption rate was not mentioned. The root development was complete, and the indication for transplantation was not mentioned. The surgical protocol was not mentioned, and the splinting method was not specified. This study did not mention orthodontic treatment, periodontal problems, or pulp necrosis [15].

This study followed different surgical protocols, but only two studies proposed their own surgical protocols. The authors used suture splinting or wire splinting as a splinting method, with a thermoplastic retainer (removable splint) as a splinting material. The duration of splinting ranged from 0.5 to 61 weeks, but it is unclear whether this duration was the same for all patients.

### 4.1. Root Development Stage

Damage to the transplants’ periodontal ligament (PDL) was the main risk factor associated with ankylosis. The tooth may typically get surrounded by a thick follicle, or PDL, during the early phases of root formation, making it simpler to remove the tooth without seriously harming the PDL. However, in fully erupted teeth, the PDL fibers are firmly attached to both the cementum and alveolar socket, and it becomes more difficult to avoid severing these fibers, which can occur at any level of the PDL. Comparatively speaking, teeth with closed apices are less likely than those with open apices to suffer adverse consequences and need to be extracted. The success rate for autotransplanting teeth with fully formed roots was found to be higher than for teeth with incomplete root formation.

Additionally, pulp necrosis and root resorption were more common in teeth with open apices. Although teeth with complete root development are frequently treated with root canal therapy to avoid periapical inflammation, which can result in more severe problems in transplants, open apices allow for better revascularization of the pulp. Endodontic autotransplantation has advanced significantly in recent years, leading to higher success rates for mature transplants [21]

### 4.2. The Autotransplanted Tooth’s Donor Site 

Ankylosis, root resorption extraction, pulp necrosis, and the greatest failure outcome were all associated with the region from which the tooth was collected for autotransplantation, which researchers examined and investigated. Apart from a modest tendency for autografts derived from the mandible to become more often ankylosed, they could not discover any meaningful correlation between the two parameters [21]

### 4.3. The Autotransplanted Tooth’s Recipient Site 

This study examined the relationship between the occurrence of unfavorable occurrences and donor tooth transplantation to the same or other places. Only one study showed a relationship between transplanting teeth to the same site and pulp necrosis, but the limited number of studies restricts safe conclusions on the matter. Ankylosis was the only unfavorable result associated with autotransplantation to a recipient site that is different from the donor site [21].

### 4.4. Donor Tooth Type—Canines vs. Molars 

When the association between certain issues and the type of donor tooth (canine or molar) was examined, it was discovered that autotransplanting molars was linked to the worst failure outcome across all trials. This result may be due to several factors. One likely reason is that the success of tooth transplantation relies on the availability of a suitable socket to accommodate the transplanted tooth, and canines are more likely to fit than molars due to their smaller size. Additionally, because multi-rooted teeth frequently have irregular root architecture, extraction of a single-rooted tooth is typically less challenging, and effective extraction depends on maintaining the periodontal membrane. Furthermore, single-rooted teeth often respond better to endodontic therapy than multi-rooted teeth do in cases of inflammatory problems. However, there is a propensity for canines to exhibit root resorption and molars to more frequently experience ankylosis [21]

### 4.5. Donor Tooth Type: 1st vs. 2nd Premolars 

The transplantation of second premolars was found to correlate with pulp necrosis. However, the validity of this finding is questionable due to the limited number of studies available. Furthermore, it is puzzling that second premolars, which ordinarily have a single root, have a higher frequency of pulp necrosis than first premolars, which often have two roots. Sadly, the study’s authors did not offer any explanation for this discovery. Based on information from two homogenous trials, it was also discovered that root resorption was related to second premolar transplants. The clinical importance of this finding is unclear, nevertheless, given the scant amount of research that is currently accessible [21].

### 4.6. The Surgical Technique Used

An analysis was conducted to determine if using a bone graft during autotransplantation surgery resulted in negative outcomes. Results demonstrated no association between the use of bone graft and the occurrence of undesirable outcomes, such as the need for extraction, pulp obliteration, root resorption, and transplant failure. According to the findings of Motoyoshi and Inoue, there was a minor tendency for the need for extraction to occur more often without the use of a bone graft [21].

### 4.7. Fixation Splint Type Used through the Healing Period 

This study also investigated whether there was a correlation between the use of a stiff or suture splint during the healing process and the incidence of unfavorable results. The findings revealed a higher incidence of ankylosis and the need for extraction in transplants that were stabilized with a suture splint. It is important to note that because of the limitation in the number of studies used in most of the analyses, it is not easy to draw definitive clinical conclusions, except for the analysis of the worst failure outcome, which was based on homogenous data from three studies [22]

### 4.8. Orthodontic Force Implementation on the Autotransplanted Teeth 

Although there was a tendency for autotransplanted teeth that did not undergo orthodontic interference to become more often ankylosed, no links between the use of orthodontic forces and unfortunate incidents were discovered. However, it is important to note that all analyses were based on only 4 or 5 studies, which limits the clinical validity of these results [22]

### 4.9. Hypermobility

Hypermobility was the only periodontal issue that could be statistically assessed using information from six studies; hence, this study focused on it. The rate of hypermobility was determined to be 8.0%, but there was significant variability among the studies included in the analysis. Sadly, because numerous researchers employed various criteria and measuring formats in the original investigations, other periodontal aspects of the transplants could not be examined in this investigation. Furthermore, the definition of hypermobility was not always consistent, which may introduce some subjectivity in the interpretation of the findings [22].

### 4.10. Pulp Necrosis 

Pulpal necrosis refers to the death of the pulp tissue in the tooth. The higher rates indicate that more teeth experienced pulp tissue death in those studies. The data from six research projects were analyzed to determine the frequencies of pulpal necrosis reported in various dental studies. The Chung et al. & Grisar et al. studies did not report any rates for pulpal necrosis [9,14]. Atala-Acevedo et al. & Machado et al. did not specify any rates for pulpal necrosis [16,17]. Almpani, Papageorgiou & Papadopoulo reported the highest incidence of pulpal necrosis at 34.3%, while the other studies either did not report any rates or reported much lower incidences, like 3.3% in the study by Rohof et al. [15,18]. 

### 4.11. Pulp Obliteration 

Pulpal obliteration refers to the complete destruction of the pulp tissue in the tooth. The reported rates indicate that around half to over half of the teeth in those studies experienced complete pulp tissue destruction. The rates of pulpal obliteration reported in the different dental studies are as follows:

- Chung et al. (2014), Grisar et al. (2018), Rohof et al. (2018), and Atala-Acevedo et al. (2016) studies did not report any rates for pulpal obliteration [9,14,15,16,17,18].
- Almpani, Papageorgiou & Papadopoulo (2015) study reported the highest rate of pulpal obliteration at 53.4% [15].
- Machado et al. (2016) study reported a rate of 57.5% for pulpal obliteration [16].
- In summary, only Almpani, Papageorgiou & Papadopoulo (2015) and Machado et al. (2016) reported rates for pulpal obliteration, which ranged from 53.4 to 57.5%. The other studies did not specify any rates [15,16].
- Machado et al. (2016) reported a slightly higher rate, so more teeth may have experienced complete pulp destruction in that study compared to Almpani, Papageorgiou & Papadopoulo (2015) [15,16].

Endodontic treatment is recommended for all autotransplanted teeth with complete root development to prevent complications such as root resorption [19,23,24,25,26,27]. Root resorption can be arrested with endodontic therapy, making it a reversible complication [28]. It is possible that root resorption has less of an effect on transplant survival and success than its frequency would suggest [29,30]. 

### 4.12. Limitations

The Umbrella review conducted a full search of electronic and manual sources to gather many relevant studies. The studies were then screened based on specific criteria related to their content and study design to ensure only high-quality studies were included in the analysis. 

Lack of uniformity made it challenging to evaluate results and draw conclusions because the chosen research had different methods, numbers of samples, and follow-up durations. It was difficult to fully comprehend the context of some studies owing to the missing data, such as the age range, indication, number of patients, and group of teeth.

The success criteria for tooth transplantation vary among the studies, with some measuring the survival rate while others measuring the success rate. This can lead to confusion and the misinterpretation of results. The studies lacked standardized reporting of the surgical protocol, splinting method, and antibiotic regimen used in the studies, making it challenging to assess the quality of the procedures. The included studies may overrepresent positive results and underrepresent negative results, leading to a biased interpretation of the effectiveness of tooth transplantation. Such potentially biased outcomes might inflate the success rate and might underrepresent the key risk factors.

During the search for relevant studies, certain databases (such as Scopus and Ovid) had limited studies available in English. Therefore, this study was restricted to “human studies only” in order to ensure data quality. Despite the initial search yielding a large number of studies, many were eliminated due to irrelevance to the subject matter, lack of an English abstract, or absence of an abstract altogether. This was performed to ensure that only high-quality studies with an international appeal were included in the final analysis. The inclusion criteria were designed to maintain the highest level of quality and similarity in the final set of studies. 

The clinical and statistical significance of the results were directly impacted by the methodological and clinical variations between the studies. The results were inconsistent and of low quality due to factors such as patient characteristics, the type of donor teeth used, the factors examined, the presentation formats of the results, the length of follow-up, the absence of a formal surgical protocol, and the lack of specific criteria for judging the success of dental autotransplantation.

## 5. Conclusions

A thorough search of the literature was conducted for the current systematic review, and certain papers were chosen for additional qualitative and quantitative analysis. Later, the data were combined, briefed, and critically assessed to form more accurate and reliable conclusions. In line with the findings of the current investigations, the process of autotransplanting teeth in humans had an estimated overall survival rate of 87.39% and an overall success rate of 90.29%. The success rate of tooth transplantation procedures varies depending on several aspects, including the patient’s age, the location of the transplanted tooth, and their overall health. Teeth transplantation procedures can be associated with several complications, including infection, rejection, and root resorption. However, this study was interpreted with some care due to the data divergence and the subpar research included in this investigation. Additional investigation is required to ascertain the effectiveness and security of tooth transplantation treatments over the long term and to pinpoint the variables that can raise their success rate and lower their danger of problems.

## Figures and Tables

**Table 1:** Assessment of the methodological quality of the included systematic reviews by Risk of Bias in Systematic Reviews (ROBIS).

Author/Year	Phase 1	Phase 2 Domain 1	Phase 2 Domain 2	Phase 2 Domain 3	Phase 2 Domain 4	Overall ROBIS Score Phase 3
Wen-Chen (2014) [14]	Yes	Low	Low	Low	Low	Low
Konstantinia Almpani (2015) [15]	Yes	Low	Low	Low	Low	Low
L.A. Machado (2016) [16]	Yes	Low	Low	Low	Unclear	Unclear
Claudia Atala-Acevedo (2016) [17]	Yes	Low	Low	Low	Low	Low
Rohof, E.C. et al. (2018) [18]	Yes	Low	Low	Low	Unclear	Unclear
Koenraad Grisar (2018) [9]	Yes	Low	Low	Low	Low	Low

**Table 2:** AMSTAR II checklist.

Author/Year	Q1	Q2	Q3	Q4	Q5	Q6	Q7	Q8	Q9	Q10	Q11	Q12	Q13	Q14	Q15	Q16
Wen-Chen (2014) [14]	**Y**	**Y**	**Y**	**PY**	**Y**	**Y**	**Y**	**Y**	**N**	**N**	**N/A**	**N**	**Y**	**Y**	**Y**	**Y**
Konstantinia Almpani (2015) [15]	**Y**	**Y**	**Y**	**Y**	**Y**	**Y**	**Y**	**Y**	**N**	**N**	**Y**	**Y**	**Y**	**Y**	**Y**	**Y**
L.A. Machado (2016) [16]	**Y**	**PY**	**N**	**PY**	**Y**	**Y**	**Y**	**Y**	**N**	**PY**	**Y**	**N**	**Y**	**Y**	**N/A**	**Y**
Claudia Atala-Acevedo (2016) [17]	**Y**	**Y**	**Y**	**Y**	**Y**	**Y**	**Y**	**Y**	**N**	**Y**	**Y**	**Y**	**Y**	**Y**	**Y**	**Y**
Rohof, E.C. et al. (2018) [18]	**Y**	**Y**	**Y**	**PY**	**Y**	**Y**	**Y**	**PY**	**N**	**Y**	**Y**	**Y**	**Y**	**Y**	**Y**	**Y**
Koenraad Grisar (2018) [9]	**Y**	**Y**	**Y**	**PY**	**Y**	**Y**	**Y**	**PY**	**N**	**N**	**Y**	**Y**	**Y**	**Y**	**Y**	**Y**

key: N = no; PY = partial yes; Y = yes; N/A = not applicable.

**Table 3:** Characteristics of the selected studies.

Author and Year	Number of Teeth	Group of Teeth	Age in Years and Gender	Donor Tooth Type	Root Formation/Development
Chung, W.C. et al. (2014) [14]	1261	Mx incisors, canine, premolars, and molars Md incisors, premolars, and molars	Not mentioned	Permanent	Complete
Almpani, K. et al. (2015) [15]	413	Canines, premolars, and molars	20 Y Both M/F	Permanent	Complete
Machado, L.A et al. (2016) [16]	274	Incisors, canines, premolars, molars, and supernumerary	9 to 46 Y Sex not mentioned	Mx and Md incisors, impacted canines, premolars, molars, and supernumerary teeth	Complete and incomplete
Atala-Acevedo, C. et al. (2016) [17]	1752	Incisors, canines, premolars, molars, and supernumerary	6.6 to 39 Y Sex not mentioned	Permanent	Not mentioned
Rohof, E.C. et al. (2018) [18]	657	Incisors, canines, premolars, and molars	7 to 29 Y Sex not mentioned	Not mentioned	Not mentioned
Grisar, K. et al. (2018) [9]	71	Not mentioned	11 to 76 Y Both M/F	Not mentioned	Complete and incomplete

**Table 4:** Data extraction from the characteristics of the included studies.

Author and Year	Number of Studies Included	Type of Study/Duration	Method of Analysis	Results
Chung, W.C. et al. (2014) [14]	26	Cohort studies and case series from 1968 to 2011	Not mentioned	Tooth autotransplantation with complete root formation is a favorable treatment with rare failure, resorption, and ankylosis rates. However, systemic antibiotics, endodontics, splinting modalities, and tooth morphology seemed to influence the outcomes.
Almpani, K. et al. (2015) [15]	28	Pragmatic clinical trails from 1983 to 2010	Relative risk	The need to extract an autotransplanted tooth seems to be on average smaller than 10%, although existing evidence precludes an accurate estimation.
Machado, L.A et al. (2016) [16]	6	Controlled trials or prospective/retrospective from 1995 to 2012	Not mentioned	The survival rate is excellent.
Atala-Acevedo, C. et al. (2016) [17]	21	Cohort studies from 1990 to 2014	Odd ratio	Overall success and survival rates were high; however, further studies are needed for the prognostic factors that influence the success of autotransplantation of teeth with an open apex
Rohof, E.C. et al. (2018) [18]	32	Cohort studies and Case Series from 1974 to 2014	Not mentioned	Both survival and success rates of autotransplantation of teeth with incomplete root formation were high (>95%), with a low rate of complications (<5%). Therefore, it could be considered a treatment option for tooth replacement
Grisar, K. et al. (2018) [9]	12	Cross-Sectional Studies and Case Series from 1983 to 2011	Not mentioned	Transplantation of maxillary canines as a legitimate treatment technique for impacted maxillary canines is difficult to treat with surgical exposure and subsequent orthodontic alignment. A good overall outcome is to be expected. There is no clear agreement, on the indications and contraindications for transplantation of maxillary canines.

**Table 5:** Surgical protocol.

Author and Year	Surgical Protocol	Splinting Method and Duration	Periodontal Problems	Antibiotic Regimen
Chung, W.C. et al. (2014) [14]	2 studies proposed their own surgical protocols (Nethandcr 1998. Gault & Warocquier- Clerout 2002) Other studies were identical or similar to the protocol, as demonstrated by Andreasen	Flexible, suture splinting, and wire splinting 0.5–61 weeks	Advanced periodontitis	Amoxicillin/clavulanic acid, tetracycline, and phenoxymethylpenicillin
Almpani, K. et al. (2015) [15]	Standardized surgical protocol	Splitting osteotomy 1 week	0 to 50% chance of the occurrence of periodontal problems	Oral antibiotics: amoxicillin, cefuroxime, and penicillin
Machado, L.A et al. (2016) [16]	Not mentioned	Not mentioned	Tooth mobility	Not mentioned
Atala-Acevedo, C. et al. (2016) [17]	Standardized surgical protocol	9 out of 21 studies mentioned splinting The duration is not mentioned	12 studies out of 21 showed periodontal problems	Not mentioned
Rohof, E.C. et al. (2018) [18]	Standardized surgical protocol	Flexible, rigid, and sutures 1 to 9 weeks	Not mentioned	21 studies out of 32 mentioned the use of systemic prophylactic antibiotics
Grisar, K. et al. (2018) [9]	Used physiological saline or intra-oral storage	Orthodontic wire splint (undefined type) 2 weeks to 12 months	Increased pocket depth and periodontal space Gingival recession	Not mentioned

**Table 6:** Long-term outcomes.

Author and Year	Success Rate	Survival Rate	Internal Resorption Rate	Ankylosis	Pulpal Necrosis
Chung, W.C. et al. (2014) [14]	Not mentioned	30.4% to 100%	0.0–75.8%	0% to 100%	Not mentioned
Almpani, K. et al. (2015) [15]	62–100%	93.48%	10.4%	6.2%	34.3%
Machado, L.A et al. (2016) [16]	57.50%	75.3% to 91%	3.4 to 3.6%	4.2% to 18.2%	13.5%
Atala-Acevedo, C. et al. (2016) [17]	89.68%	98.21%	0% to 2l.3%	0 to 20%	Not mentioned
Rohof, E.C. et al. (2018) [18]	96.60%	After 10 years >96.3%	2.9%	2%	3.3%
Grisar, K. et al. (2018) [9]	Not mentioned	62-100%	3–76%	23.8%	Not mentioned

## Data Availability

The datasets used and analyzed during the current study are available from the corresponding author on reasonable request.

## References

[1] Malmgren B., Andreasen J.O., Andreasen J.O., Malmgren B., Bakland L.K., Flores M.T. (2019). Tooth autotransplantation: An overview of techniques and therapeutic concepts. Textbook and Color Atlas of Traumatic Injuries to the Teeth.

[2] Pohl Y., Filippi A., Kirschner H., Pohl T. (2014). Tooth autotransplantation: An overview for clinical practice. Swiss Dent. J..

[3] Zhang W., Walboomers X.F., Shi S., Fan M., Jansen J.A. (2006). Multilineage differentiation potential of stem cells derived from human dental pulp after cryopreservation. Tissue Eng..

[4] Nimčenko T., Omerca G., Varinauskas V., Bramanti E., Signorino F., Cicciù M. (2013). Tooth auto-transplantation as an alternative treatment option: A literature review. Dent. Res. J..

[5] Yanik L., Aksoy A., Tugsel Z., Isiksal E. (2012). Long-term evaluation of teeth transplantation: A case series. Int. J. Oral Maxillofac. Surg..

[6] Kirsch J.M., Wiskott A., Reichert T.E., Wolfart S. (2009). Teeth transplantation—A systematic review. Dent. Traumatol..

[7] Park J.H., Kim Y.G., Suh J.Y., Jin M.U., Lee J.M. (2022). Long-Term Survival Rate of Autogenous Tooth Transplantation: Up to 162 Months. Medicina.

[8] Muhamad A.-H., Nezar W., Mai A., Azzaldeen A. (2016). Tooth Autotransplantation; Clinical Concepts. IOSR J. Dent. Med. Sciences..

[9] Grisar K., Chaabouni D., Romero L.P.G., Vandendriessche T., Politis C., Jacobs R. (2018). Autogenous transalveolar transplantation of maxillary canines: A systematic review and meta-analysis. Eur. J. Orthod..

[10] Kakde K.K.R. (2022). Tooth Autotransplantation as an Alternative Biological Treatment: A Literature Review. Cureus.

[11] Choi S.C., Kwon Y.D., Kim K.C., Kim G.T. (2010). The Effects of Topical Application of Bisphosphonates on Replanted Rat Molars. Dent. Traumatol..

[12] Shea B.J., Grimshaw J.M., Wells G.A., Boers M., Andersson N., Hamel C., Bouter L.M. (2007). Development of AMSTAR: A measurement tool to assess the methodological quality of systematic reviews. BMC Med. Res. Methodol..

[13] Liberati A., Altman D.G., Tetzlaff J., Mulrow C., Gøtzsche P.C., Ioannidis J., Clarke M., Devereaux P., Kleijnen J., Moher D. (2009). The PRISMA statement for reporting systematic reviews and meta-analyses of studies that evaluate health care interventions: Explanation and elaboration. J. Clin. Epidemiol..

[14] Chung W.-C., Tu Y.-K., Lin Y.-H., Lu H.-K. (2014). Outcomes of autotransplanted teeth with complete Root Formation: A systematic review and meta-analysis. J. Clin. Periodontol..

[15] Almpani K., Papageorgiou S.N., Papadopoulos M.A. (2015). Autotransplantation of teeth in humans: A systematic review and meta-analysis. Clin. Oral Investig..

[16] Machado L.A., Do Nascimento R.R., Ferreira D.M., Mattos C.T., Vilella O.V. (2016). Long-term prognosis of tooth autotransplantation: A systematic review and meta-analysis. Int. J. Oral Maxillofac. Surg..

[17] Atala-Acevedo C., Abarca J., Martínez-Zapata M.J., Díaz J., Olate S., Zaror C. (2016). Success rate of autotransplantation of teeth with an open apex: Systematic review and meta-analysis. J. Oral Maxillofac. Surg..

[18] Rohof E.C., Kerdijk W., Jansma J., Livas C., Ren Y. (2018). Autotransplantation of teeth with incomplete Root Formation: A systematic review and meta-analysis. Clin. Oral Investig..

[19] Nethander G., Andersson J.E., Hirsch J.M. (1988). Autogenous free tooth transplantation in man by a 2-stage operation technique. A longitudinal intra-individual radiographic assessment. Int. J. Oral Maxillofac. Surg..

[20] Gault P.C., Warocquier-Clerout R. (2002). Tooth auto-transplantation with double periodontal ligament stimulation to replace periodontally compromised teeth. J. Periodontol..

[21] Motoyoshi M., Inoue S. (2015). Risk factors for failure of tooth autotransplantation: A retrospective study. Oral Surg. Oral Med. Oral Pathol. Oral Radiol..

[22] Ramassamy E. (2023). Perspective Chapter: Splinting of Traumatized Teeth. Dental Trauma and Adverse Oral Conditions—Practice and Management Techniques.

[23] Azaz B., Lustmann J., Shaharabany M., Shapira J. (1978). Autotransplantation of teeth. Oral Surg. Oral Med. Oral Pathol..

[24] Kahnberg K.E. (1987). Autotransplantation of teeth (I). Indications for transplantation with a follow-up of 51 cases. Int. J. Oral Maxillofac. Surg..

[25] Andreasen J.O., Paulsen H.U., Yu Z., Schwartz O. (1990). A long-term study of 370 autotransplanted premolars. Part III. Periodontal healing subsequent to transplantation. Eur. J. Orthod..

[26] Lundberg T., Isaksson S. (1996). A clinical follow-up study of 278 autotransplanted teeth. Br. J. Oral Maxillofac. Surg..

[27] Arikan F., Uzun B., Evlioglu G., Malkoc S. (2008). Evaluation of prognostic factors in autotransplantation of teeth. Oral Surg. Oral Med. Oral Pathol. Oral Radiol. Endodontol..

[28] Ingle J., Glick D.H. (1965). Modern Endodontic Therapy.

[29] Robinson P.J., Guernsey L.H. (1980). Clinical Transplantation in Dental Specialties.

[30] Kind V., Strbac G.D., Franz A., Türp J.C., Schedle A. (2010). Survival and success rates of autotransplanted premolars: A long-term follow-up of up to 20 years. J. Oral Maxillofac. Surg..

